# A multicriteria model for prioritizing 5G network deployment with Monte Carlo stability analysis: A case study in Magdalena, Colombia

**DOI:** 10.1371/journal.pone.0334781

**Published:** 2025-10-17

**Authors:** Ronald Martínez Abuabara, Carlos Arturo Robles, Luis Leonardo Camargo

**Affiliations:** 1 Faculty of Engineering, Universidad del Magdalena, Santa Marta, Magdalena, Colombia; 2 GIDEAM – Research Group on Electronic Development and Mobile Applications, Universidad del Magdalena, Santa Marta, Magdalena, Colombia; 3 MAGMA Ingeniería – Research Group on Applied Mathematics for Engineering, Universidad del Magdalena, Santa Marta, Magdalena, Colombia; Opole University of Technology: Politechnika Opolska, POLAND

## Abstract

The global deployment of 5G mobile networks has followed a progressive and strategic approach, depending on regional characteristics. This study develops a reference framework to prioritize areas for deployment, considering technical, sociodemographic, geographic, and economic criteria. The methodology integrates multi-criteria decision-making techniques such as AHP, CRITIC, TOPSIS, and SAW to evaluate and prioritize alternatives. This framework was applied to the municipalities of the Magdalena department in Colombia. The AHP results indicate that technical criteria are the most relevant in the selection process, with a weight of 34.3%, followed by sociodemographic criteria at 33.6%, geographic criteria at 19.47%, and economic criteria at 12.63%. A high similarity in municipality prioritization was observed, with a correlation of *ρ* = 0.9897 according to Spearman’s coefficient. Using TOPSIS and SAW, the municipality of Ciénaga ranks first, given that the sub-criteria of population size, area, and 4G coverage hold the highest relevance in the selection process, with percentages of 13.13%, 12.16%, and 11.12%, respectively, while the municipalities of Fundación and Plato alternate between second and third place. On the other hand, La Zona Bananera ranks fourth and fifth. To assess the model’s robustness against variability in the criterion weights, a sensitivity analysis was conducted using the Monte Carlo method with 10,000 iterations. The results indicate that the ranking remains stable, with an average correlation of *ρ* = 0.9010 between the rankings obtained with SAW and the final ranking using TOPSIS. The influence of high-weight and highly correlated sub-criteria was also assessed. Rankings from AHP-SAW and AHP-TOPSIS were compared with CRITIC-SAW and CRITIC-TOPSIS, yielding correlations of 0.98 and 0.76, respectively. It can be concluded that the deployment of 5G networks can be systematically prioritized based on the mentioned criteria, using a model that remains stable despite changes in criterion weights.

## Introduction

Fifth-generation (5G) mobile networks represent a major advancement in telecommunications, offering data transfer speeds of up to 5 Gbps, latencies below 1 ms, and connectivity for up to one million devices per square kilometer. These capabilities enable advanced applications across various industries through three key scenarios: Enhanced Mobile Broadband (eMBB), Massive Machine-Type Communications (mMTC), and Ultra-Reliable Low-Latency Communications (URLLC) [1].

5G technology is poised to transform key sectors such as healthcare, education, and industrial automation. Its service scenarios create new opportunities for the development of smart cities and autonomous, virtual, and intelligent systems. Studies have shown that 5G expansion drives global competitiveness by enabling immersive applications such as virtual and augmented reality, the Internet of Things (IoT), and telemedicine, ultimately helping to reduce inequalities and improve quality of life [1,2]. However, the implementation of 5G presents technical, economic, and regulatory challenges that vary depending on each country’s geographic and socioeconomic context.

The deployment of 5G has followed different strategic approaches. In some countries, priorities have included revenue generation, maximizing digital inclusion, early infrastructure rollout, ensuring both rural and urban coverage, or focusing on major cities. Despite these variations, all strategies share common goals: reducing the digital divide, enhancing economic competitiveness, and improving consumer welfare. To achieve these objectives, many countries have implemented spectrum allocation processes through licensing auctions. The following section outlines key aspects of these processes in selected Latin American countries.

Chile has prioritized infrastructure investments through public auctions. Five spectrum auctions have been conducted: four in 2020, one in 2021, and another in 2024, allocating spectrum in the 700 MHz, 3.5 GHz, and 26 GHz bands. The auction agreements include requirements such as achieving 90% coverage within a maximum period of three years and near-total coverage of municipalities in the 700 MHz and AWS (Advanced Wireless Services) bands. Additionally, they mandate service provision in 366 small localities, 65 routes, approximately 200 healthcare facilities, 24 ministries, regional and provincial capitals, airports, aerodromes, scientific research centers, higher education institutions, and seaports. By 2023, only 15% of mobile connections in Chile operated on 5G networks. By 2024, some operators had failed to meet their infrastructure deployment plans, impacting national development [3,4].

Brazil has adopted a phased approach to optimize spectrum distribution through a multiband auction, allocating spectrum in the 2.3 GHz, 3.5 GHz, and 26 GHz bands. Previously, following the analog switch-off, the 700 MHz band had been auctioned for 4G networks. To encourage investment, the General Telecommunications Law introduced incentives for operators, including extending license terms to 20 years with automatic renewal contingent on compliance with coverage and service obligations. Additionally, operators were allowed to make annual payments for spectrum acquisition throughout the license period, while infrastructure investments were required to represent up to 94% of the total auctioned value. Furthermore, auction bids exceeding the reserve price could be converted into voluntary investment commitments. As a result of these measures, by 2023, 9% of mobile connections in Brazil operated on 5G networks, with coverage primarily concentrated in major cities, reaching 60% of the population by 2024 [3,5].

In Colombia, the spectrum allocation process began in 2019 with the assignment of the 700 MHz, 1.9 GHz, and 2.5 GHz bands for 4G networks and their evolution. As part of this auction, coverage commitments were established for rural areas over a five-year period. By late 2023, a new auction was conducted, including the 3.5 GHz band along with additional spectrum in the 700 MHz, 1.9 GHz, extended AWS, and 2.5 GHz bands, intended for 5G network deployment [6]. This auction introduced seven-year deployment obligations, with annual targets for the number of 5G base stations per capita, differentiated by city type and population size. Additionally, minimum 4G coverage requirements were set for specific highways, along with broadband internet service commitments via fiber optics for certain educational institutions. As of June 2024, Colombia had deployed 1,167 5G base stations nationwide [7].

Regardless of the country, strategy, or commitments undertaken, 5G network deployment is a gradual process spanning several years. Operators must prioritize base station locations to ensure adequate coverage and network implementation in each region, aligning with contractual auction obligations and the area’s social and economic characteristics.

In Colombia, 5G network implementation follows specific targets. In the capital city, Bogotá, by 2025, there should be one base station for every 80,000 inhabitants, with an annual increase in deployment until reaching one base station per 40,000 inhabitants by 2030. In the capital cities of the country’s 32 departments, at least 50% are expected to have one base station per 80,000 inhabitants by 2025, with full coverage across all department capitals by 2026, and an increase to one base station per 40,000 inhabitants by 2030. In municipalities with more than 200,000 inhabitants, operators must provide coverage by 2027, ensuring one base station per 60,000 inhabitants, scaling up to one per 40,000 inhabitants by 2030. For other municipalities not explicitly defined, deployment depends on market supply and demand, as there are no specific regulatory obligations for operators [6].

Despite these targets, connectivity remains a challenge in departments located far from major urban centers, underscoring the need for strategic planning to ensure equitable coverage, particularly in regions with diverse geographical and socio-economic conditions. This is the case for the Magdalena Department, which features irregular topography and pronounced socio-economic disparities among its municipalities, complicating the optimal planning of 5G deployment.

In this context, Multi-Criteria Decision Making (MCDM) serves as a crucial tool for evaluating and balancing technical, economic, and social factors. Methods such as the Analytic Hierarchy Process (AHP), the Technique for Order Preference by Similarity to Ideal Solution (TOPSIS), and Simple Additive Weighting (SAW) have proven effective in prioritizing infrastructure projects. These approaches enable the weighted evaluation of criteria, facilitating the selection of optimal alternatives [8–10]. By integrating quantitative and qualitative data, MCDM significantly enhances 5G network planning, ensuring a robust and objective decision-making process.

Previous studies have applied multi-criteria methods to telecommunications network planning. The work by [11] presents a decision-making framework for deploying critical telecommunications infrastructure. It compares different implementation strategies—commercial, hybrid, and dedicated networks—based on criteria such as coverage, traffic capacity, availability, security, and cost.

The study by [12] introduces an MCDM approach designed for high-uncertainty environments using Pythagorean Fuzzy Sets (PFS). It combines the Combined Compromise Solution (CoCoSo) method with the Criteria Importance Through Inter-criteria Correlation (CRITIC) method to develop a model applied to the 5G industry. This model evaluates leading companies in the sector—Huawei, ZTE, Nokia, Ericsson, and Samsung—based on key criteria such as maximum data rate, spectral efficiency, connection density, and latency.

Similarly, the research conducted by [13] proposes a risk assessment framework for 5G wireless equipment construction projects, using a case study in Nanjing, China. The study employs the Literature-Delphi method to define selection criteria, integrates AHP and information entropy to weight risk factors, and applies an enhanced fuzzy evaluation method to quantify project-specific risks. A total of 27 risk indicators were identified across four categories: Technical Risk, Environmental Risk, Economic and Management Risk, and Planning Risk.

The study by [14] applies AHP and TOPSIS to evaluate and rank service areas requiring 5G upgrades within a telecommunications company in Turkey. This methodology assigns weights to criteria such as bandwidth usage, signal quality, the number of users with 5G-compatible terminals, and the area’s road and building infrastructure.

Research presented in [15] proposes an advanced method for resource allocation decision-making in 5G networks using fuzzy logic. The most notable feature is its incorporation of uncertainty and subjective expert opinions through fuzzy trigonometric operators, improving analysis accuracy in complex scenarios. This approach offers valuable insight into how classic methods like AHP or TOPSIS can be extended to better handle imprecise inputs.

Article [16] develops a hybrid model to assess overall 5G base station performance by combining the Bayesian Best-Worst Method (Bayesian BWM) with the DQ-GRA technique. This approach enables more consistent criterion prioritization, even under uncertainty or limited data, and accurately ranks alternatives. Its main contribution lies in demonstrating how advanced weighting and analysis methods can enhance technical evaluation in planning processes.

Study [17] presents an optimal resource allocation approach in 5G networks based on the combination of the PROMETHEE-II method and a Stochastic Learning with Exploration (SLE) strategy. PROMETHEE-II prioritizes network slicing requests based on multiple criteria, while SLE dynamically adjusts allocation in real time. Results show improvements in request acceptance and reduced resource waste compared to traditional algorithms, demonstrating applicability in differentiated 5G services.

In [18], a remote patient monitoring system is developed that integrates Artificial Neural Networks (ANN) for biomedical data analysis and a hybrid model using Choquet fuzzy VIKOR for clinical decision prioritization. This approach considers interdependence between physiological criteria and allows decision-making under uncertainty. Blockchain-based security is also incorporated to protect data transmitted over 5G networks, demonstrating usefulness in telehealth with high reliability and low latency.

Finally, [19] applies fuzzy AHP to determine optimal locations for road units in vehicular networks, considering factors such as traffic and road conditions. The key insight is that the criteria are adapted to the physical context essential in regions with diverse terrain like Magdalena. This reinforces the idea that decision models must account for both technical and territorial realities to enable effective planning.

Considering the prior context and reviewed literature, the contribution of this work does not lie in inventing a new algorithm, but in rigorously applying a validated multicriteria framework that integrates technical, social, and productive criteria to prioritize 5G network deployment in a specific context like the Department of Magdalena, which features terrain irregularities and socio-productive differences in each municipality, where each one demands differentiated telecommunications services. The study compares AHP-TOPSIS and AHP-SAW and incorporates a Monte Carlo-based stability analysis and a dependency analysis to evaluate the robustness of the rankings under uncertainty. Its application not only optimizes 5G infrastructure investment and reduces the digital connectivity gap but also provides a replicable tool for formulating public policies and implementing more efficient and equitable deployment strategies in Colombia and other regions with similar conditions.

## Materials and methods

**Fig 1:**
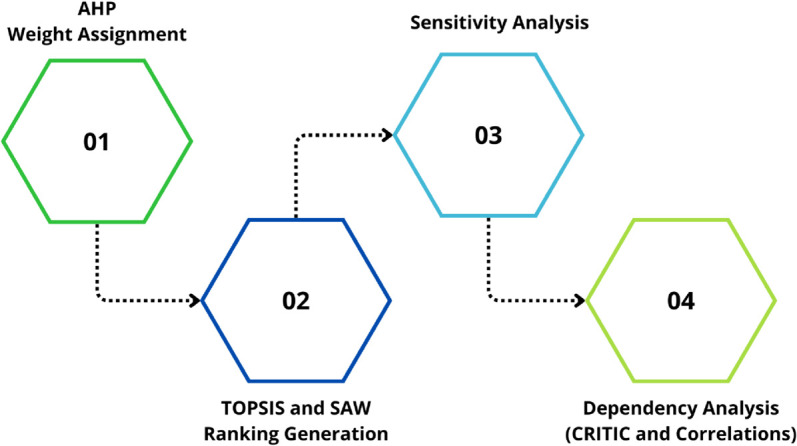
Stages of the methodology for prioritizing 5G deployment.

The methodology adopted in this study was structured into four stages. In the first stage, the Analytic Hierarchy Process (AHP) was applied to determine the weights of the criteria and subcriteria. The second stage involved the use of the TOPSIS and SAW techniques to generate a ranking of alternatives based on the previously assigned weights. In the third stage, a sensitivity analysis was conducted to assess the stability of the model with respect to variations in the criteria and subcriteria. Finally, in the fourth stage, a dependency analysis was performed, considering correlations among criteria and applying the CRITIC method as a complementary validation mechanism. Fig 1 schematically summarizes the main stages of the methodology implemented in this study.

### Application of AHP

The Analytic Hierarchy Process (AHP), introduced by Saaty, is a structured decision-making method that decomposes complex problems into a hierarchical model consisting of a goal, criteria, subcriteria, and alternatives. This hierarchical structure provides a systematic and graphical framework for organizing, analyzing, and prioritizing information. AHP allows for pairwise comparisons between criteria and subcriteria, enabling their weighting at each hierarchical level. The method integrates both quantitative and qualitative data using Saaty’s fundamental scale and includes a consistency check to ensure reliable judgments. Additionally, sensitivity analysis is conducted to assess the impact of changes in criteria weights on the final decision [8,20,21].

**Fig 2:**
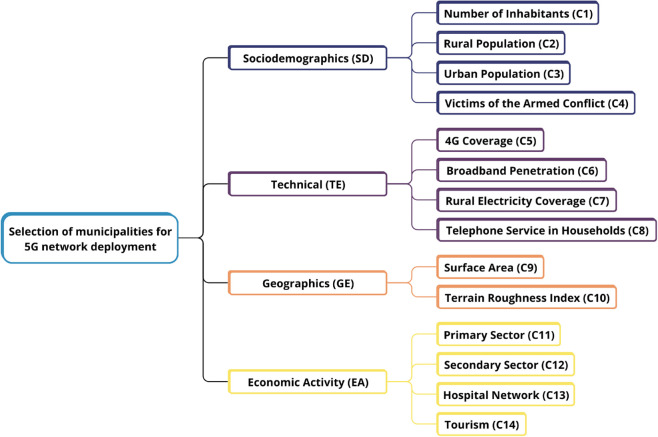
Hierarchical structure of AHP selection process.

As an initial step, the criteria and subcriteria for prioritizing the alternatives (municipalities) were identified based on a comprehensive literature review and expert consultations. The selected criteria were required to be mutually exclusive to avoid redundancy. Although AHP assumes independence between criteria, this assumption was critically reviewed considering potential correlations (e.g., between population size and economic activity). However, interdependence was not formally modeled in this study to preserve methodological consistency with the AHP framework and to facilitate the pairwise comparison process by experts. The validity of this assumption was later examined through a correlation analysis between the selected criteria, as presented in the Results section [22]. Fig 2 presents the hierarchical structure used, followed by a detailed description of each subcriterion.

**Sociodemographic Criteria (SD)**

- **Number of Inhabitants (C1):** Total population residing in the municipality, which allows estimating the potential demand for 5G services, facilitating investment decisions with greater social and economic impact.
- **Rural Population (C2):** Number of individuals living in rural areas outside the urban center, and its consideration is key to identify connectivity gaps in historically marginalized areas.
- **Urban Population (C3):** Number of individuals residing in the municipality’s urban area; its importance lies in the fact that these areas concentrate a higher density of users and services, which favors the efficiency in the deployment of 5G infrastructure.
- **Victims of the Armed Conflict (C4):** Number of individuals affected by the Colombian internal conflict, including displaced persons, injured individuals, or those who have suffered violence or property loss. It is a relevant criterion for prioritizing regions that require social reconstruction and access to development-enabling technologies.

**Technical Criteria (TE)**

- **4G Coverage (C5):** It refers to the availability of fourth-generation (4G) mobile network technology in the municipality, which reflects the technical readiness of the territory and allows for a more efficient transition to 5G technology.
- **Broadband Penetration (C6):** Proportion of households, businesses, or users with broadband internet access, being a key indicator of the level of local technological adoption and the potential for effective use of new networks.
- **Rural Electricity Coverage (C7):** Proportion of the municipality’s rural area that has access to the electrical grid compared to the total rural area. This essential technical condition is considered important for the installation and operation of base stations in remote areas.
- **Telephone Service in Households (C8):** Percentage of households with access to any type of telephone service, which serves as an indirect indicator of the basic level of connectivity and makes it possible to detect areas with urgent need for improvement.

**Geographic Criteria (GE)**

- **Surface Area (C9):** Total land area of the municipality, measured *Km*^2^. Its inclusion is essential since larger areas require more infrastructure to ensure effective coverage.
- **Terrain Rugosity Index (C10):** Measure of elevation variability within the municipality. It is crucial to anticipate technical difficulties in signal propagation.

**Economic Activity Criteria (AE)**

- **Primary Sector (C11):** Percentage of economic activities related to the extraction and transformation of natural resources, including agriculture, livestock, forestry, and fishing. Including this criterion allows identifying municipalities with a productive rural vocation that can significantly benefit from access to 5G technologies to improve productivity.
- **Secondary Sector (C12):** Percentage of economic activities focused on manufacturing and industrial production, including food processing, textiles, chemicals, and machinery. This criterion reflects the industrial potential of the municipality and its ability to leverage 5G-enabled solutions such as automation, real-time monitoring and Industrial Internet of Things (IoT), which can enhance local competitiveness.
- **Hospital Network (C13):** Classification of hospitals into three levels of care—high (2), medium (1), and low (0)—based on the complexity and capacity of medical services. This classification was made according to Resolution No. 3100 of 2019 from the Ministry of Health and Social Protection [23]. This criterion allows prioritizing municipalities where the 5G network can have a more direct impact on the improvement of health services through solutions such as telemedicine, remote patient management and hospital digitalization.
- **Tourism (C14):** Number of registered tourism accommodation establishments in the municipality, according to the Colombian National Tourism Registry. Municipalities with tourism activity can benefit from the deployment of 5G networks to offer digital visitor services and improve the competitiveness of the destination, boosting the local economy.

Following the identification of criteria and subcriteria, a prioritization process was conducted using the AHP method. This approach structures the decision-making process through comparison matrices, where decision-makers or experts provide pairwise judgments without knowing the exact weight of each criterion or subcriterion in advance [24].

AHP is based on the following axioms: *Axiom 1:* Presumption of reciprocal judgments – If A is a pairwise comparison matrix, then the condition $a_{ij}=1/a_{ji}$ holds; *Axiom 2:* Requirement of elemental homogeneity – The elements being compared belong to the same hierarchy or order of magnitude; *Axiom 3:* Existence of a dependent or hierarchical structure – The components of two successive levels are hierarchically dependent on each other; and *Axiom 4:* Expectation of hierarchical order – The structure must reflect expectations regarding criteria and alternatives [21,25].

Let ***A*** be an *n×n* matrix, where each element *a*_*ij*_ (for i,j=1,2,…,n) represents the pairwise comparison of criteria or subcriteria. The value of *a*_*ij*_ expresses the relative preference of criterion *i* over criterion *j*, as defined in Eq (1).

$$A=\begin{array}{c} A_{1}\\ A_{2}\\ \vdots \\ A_{n} \end{array}[\begin{array}{cccc} a_{11} & a_{12} & \ldots & a_{1n}\\ a_{21} & a_{22} & \ldots & a_{2n}\\ \vdots & \vdots & \ddots & \vdots \\ a_{n1} & a_{n2} & \ldots & a_{nn} \end{array}]$$ (1)

When *i* = *j*, the value *a*_*ij*_ is equal to 1, since a criterion is always equal to itself in the comparison matrix. Consequently, under the reciprocity assumption in the pairwise comparison matrix (if the decision maker judges criterion *A* to be *k* times more important than criterion *B*, then *B* is 1/*k* times as important as *A*) it follows that $a_{ij}a_{ji}=1$ holds, as shown in Eq (2). This reciprocity assumption, although common in AHP, is not always satisfied in practice by decision makers [26].

$$A=[\begin{array}{cccc} 1 & a_{12} & \ldots & a_{1n}\\ 1/a_{12} & 1 & \ldots & a_{2n}\\ \vdots & \vdots & \ddots & \vdots \\ 1/a_{1n} & 1/a_{2n} & \ldots & 1 \end{array}]$$ (2)

To compute *a*_*ij*_ eight experts from the academic, productive, and territorial transformation sectors were consulted. A preference scale ranging from 1 to 9, with corresponding reciprocal values, was employed according to Saaty’s scale [27,28], as shown in Table 1.

**Table 1:** Saaty’s scale for pairwise comparison process.

Numeric Value	Description
1	Equally preferable
2	Between equally and moderately preferable
3	Moderately preferable
4	Between moderately and strongly preferable
5	Strongly preferable
6	Between strongly and very strongly preferable
7	Very strongly preferable
8	Between very strongly and extremely preferable
9	Extremely preferable

Source: Adapted from [28].

**Fig 3:**
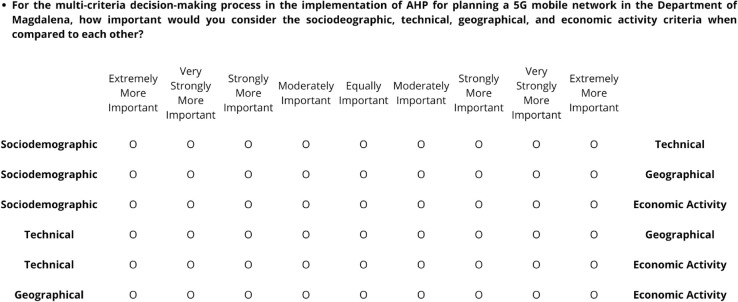
Excerpt from the survey sent to experts.

To systematize the expert consultation process, a structured survey was designed to facilitate pairwise comparisons among criteria and subcriteria. This resulted in a survey with five comparison tables. Fig 3 illustrates an excerpt from the survey used for the criteria assessment.

To validate the expert judgments, the Consistency Ratio (CR) was calculated [29]. This required solving the eigenvalue problem $\text{A}*\text{w}=\lambda_{max}*w$, where ***A*** is the pairwise comparison matrix, ***w*** its principal (normalized) eigenvector, and $\lambda_{max}$ represents the matrix’s largest eigenvalue.

The Consistency Index (CI) is then computed using Eq (3):

$$CI=\left(\frac{\lambda_{max}-n}{n-1}\right)$$ (3)

where *n* is the number of elements in the comparison matrix.

*CR* is determined by dividing *CI* by the Random Consistency Index (RI), as shown in Eq (4).

$$CR=\frac{CI}{RI}$$ (4)

*RI* values are obtained from random reciprocal pairwise comparison matrices generated by Saaty for different matrix orders *n*, as presented in Table 2.

**Table 2:** Random Consistency Index for different matrix sizes.

N	1	2	3	4	5	6	7	8	9	10	11	12
RI	0.00	0.00	0.52	0.89	1.11	1.25	1.35	1.40	1.45	1.49	1.52	1.54

Source: Adapted from: [30]

If CR≤0.1, the consistency of the comparisons is acceptable; otherwise, the expert must revise their judgments to improve consistency.

With the validated weights determined through AHP, the methodology proceeds to the second phase, where the prioritization of alternatives is performed using TOPSIS and SAW.

### Application of TOPSIS and SAW

This section describes the implementation of TOPSIS and SAW for prioritizing municipalities, detailing the type and source of the data used for each subcriterion in the ranking process.

#### Systematization of TOPSIS.

TOPSIS, introduced by Hwang and Yoon [9], facilitates decision-making by systematically structuring and simplifying the evaluation of multiple alternatives [31]. The method defines:

Given a set of alternatives *A*_*i*_, where i=1,2,…,m, a set of criteria *C*_*j*_ where j=1,2,…,n, the criteria weights *w*_*j*_, and a decision matrix where the values *x*_*ij*_ represent the direct measurements of each alternative with respect to the defined criteria. To ensure a consistent orientation of all criteria, a normalization process is applied, so that benefit criteria are maximized and cost criteria are minimized, as required by the logic of the TOPSIS method. This process allows the alternatives to be compared within a unified evaluation framework.

A positive ideal solution *A*^ + ^ is defined as the best possible choice, while a negative ideal solution *A*^−^ represents the least desirable option. It is important to note that the decision-maker always aims to choose *A*^ + ^, and if no available alternative exactly matches this ideal solution, the closest option is chosen instead.

To implement TOPSIS, an algorithm was developed in Python, enabling efficient and systematic calculations. This approach fully automates the normalization of the decision matrix, the weighting of criteria, the computation of distances to ideal solutions, and the generation of the final ranking of alternatives. Additionally, the use of specialized libraries such as NumPy and Pandas optimizes data processing and ensures the reproducibility of results.

**Construction of the decision matrix.**

This was developed based on the m alternatives *A*_*i*_, where i=1,2,…,m, which will be evaluated according to the given criteria *C*_*j*_, where j=1,2,…,n as shown in Table 3).

**Table 3:** Decision matrix based on alternatives and criteria.

	*w*_1_	*w*_2_	…	*w*_*n*_
	*C*_1_	*C*_2_	…	*C*_*n*_
*A*_1_	*x*_11_	*x*_12_	…	*x*_1*n*_
*A*_2_	*x*_21_	*x*_22_	…	*x*_2*n*_
⋮	⋮	⋮	⋱	⋮
*A*_*m*_	*x*_*m*1_	*x*_*m*2_	…	*x*_*mn*_

Source: Adapted from: [32]

Where *x*_*ij*_ represents the evaluation of alternative *A*_*i*_ concerning criterion *C*_*j*_, and $W=[w_{1},w_{2},\ldots ,w_{n}]$ is the vector of criterion weights.

**Normalization and weighting of the decision matrix.**

Since the elements of the matrix may belong to different domains, normalization is required. The normalization process is conducted using Eq (5):

$$n_{ij}=\frac{x_{ij}}{\sqrt{\sum_{j=1}^{m}(x_{ij}{)}^{2}}}\;(j=1,2,\ldots ,n;i=1,2,\ldots ,m)$$ (5)

To construct the weighted normalized decision matrix, Eq (6) is used, where *w*_*j*_ represents the weight assigned to each specific criterion.

$$v_{ij}=w_{j}*n_{ij}\;(j=1,2,\ldots ,n;i=1,2,\ldots ,m)$$ (6)

Where *w*_*j*_ is the weight assigned to each criterion.

**Estimation of the Positive Ideal Solution (PIS) and Negative Ideal Solution (NIS).**

The positive ideal solution (*A*^ + ^) and negative ideal solution (*A*^−^) are computed using Eqs (7) and (8), respectively.

$$A^{+}=\{{A_{1}}^{+},{A_{2}}^{+},\ldots ,{A_{j}}^{+}\},A_{j}^{+}=\{(max(v_{ij})\forall j\in J)\vee (min(v_{ij})\forall j\in J^{\prime })\}$$ (7)

$$A^{-}=\{{A_{1}}^{-},{A_{2}}^{-},\ldots ,{A_{j}}^{-}\},A_{j}^{-}=\{(min(v_{ij})\forall j\in J)\vee (max(v_{ij})\forall j\in J^{\prime })\}$$ (8)

Where *J* represents benefit criteria and $J^{\prime }$ represents cost criteria.

**Estimation of distance measures.**

To determine the distance of each alternative from the positive ideal solution *d*^ + ^ and the negative ideal solution *d*^−^, Eqs (9) and (10) are used:

$${d_{i}}^{+}=\sqrt{\sum_{j=1}^{n}(v_{ij}-{v_{j}}^{+}{)}^{2}}\;(i=1,2,\ldots ,m)$$ (9)

$${d_{i}}^{-}=\sqrt{\sum_{j=1}^{n}(v_{ij}-{v_{j}}^{-}{)}^{2}}\;(i=1,2,\ldots ,m)$$ (10)

**Calculation of relative closeness to the ideal solution.**

The relative closeness *R*_*i*_ to the positive ideal solution is computed using Eq (11). The closer *R*_*i*_ is to one, the nearer the *i-th* alternative is to the ideal solution.

$$R_{i}=\frac{{d_{i}}^{-}}{{d_{i}}^{+}+{d_{i}}^{-}}\;(i=1,2,\ldots ,m)$$ (11)

**Ranking of alternatives.**

The alternatives are ranked in descending order of *R*_*i*_, starting with the one that is closest to the ideal solution (i.e., with the highest relative closeness), meaning it is nearer to the positive ideal solution and farther from the negative ideal solution.

**Fig 4:**
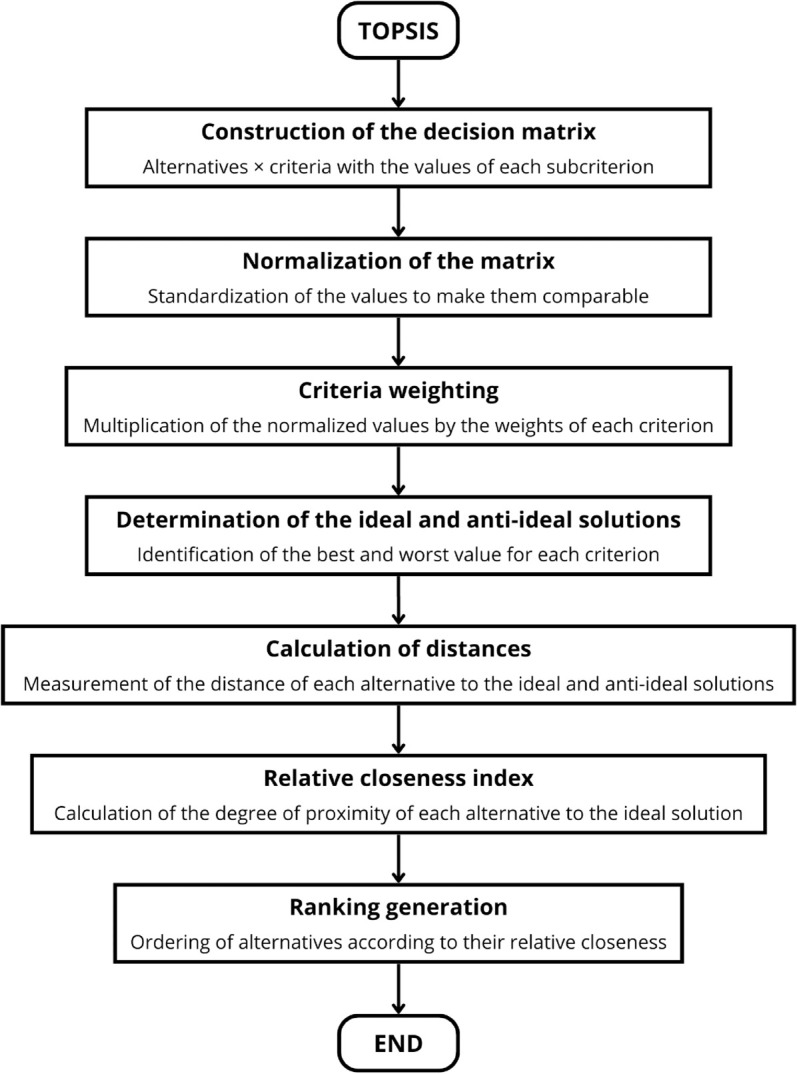
Methodological scheme of the TOPSIS method.

In summary, the TOPSIS method allows the evaluation of alternatives by simultaneously considering their proximity to the ideal solution and their distance from the anti-ideal solution, ensuring an objective ranking consistent with the weights assigned to the criteria. The TOPSIS methodology is schematically summarized in Fig 4.

#### Systematization of SAW.

The Simple Additive Weighting (SAW) method is one of the most straightforward and widely used decision-making techniques, based on the direct sum of weighted scores assigned to each alternative across all criteria [33].

Similar to the TOPSIS method, SAW was implemented using a Python algorithm with libraries such as NumPy and Pandas. The process involved constructing the decision matrix, normalizing the data, and ultimately calculating the scores for the alternatives.

The decision matrix was structured following the same principle shown in Table 3. Data normalization depends on whether the criteria correspond to benefits or costs, applying Eqs (12) and (13), respectively.

$$R_{ij}=\frac{x_{ij}}{max(x_{ij})}$$ (12)

$$R_{ij}=\frac{min(x_{ij})}{x_{ij}}$$ (13)

The weighted score for each alternative is calculated using Eq (14).

$$S_{i}=\sum_{j=1}^{m}w_{j}*R_{ij}$$ (14)

Where *S*_*i*_ is the total score of alternative *A*_*i*_, *w*_*j*_ is the weight of criterion *j*, and *R*_*i*_*j* is the normalized value of alternative *i* for criterion *j*.

**Fig 5:**
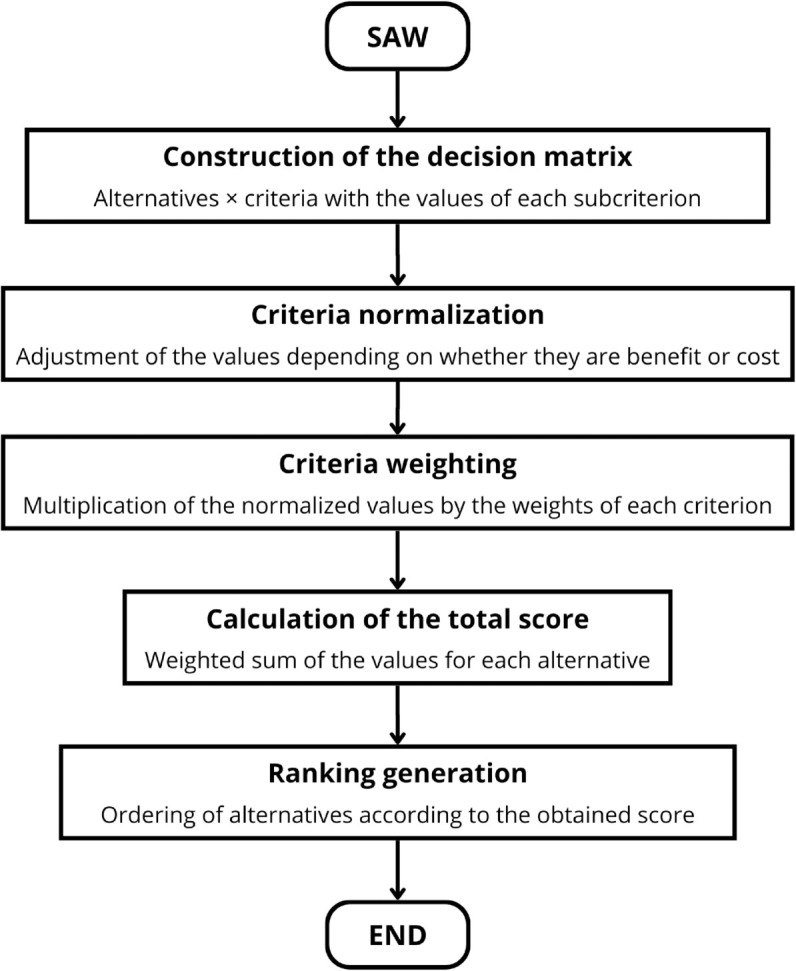
Methodological scheme of the SAW method.

Finally, the alternatives are ranked based on their total score, with the option having the highest *S*_*i*_ being the most recommended. The steps comprising this process are illustrated in the schematic diagram in Fig 5, which graphically summarizes the SAW methodology.

#### Data collection, computation, and processing.

To apply TOPSIS and SAW, the 29 municipalities of the Magdalena Department were defined as the set of alternatives. Subsequently, data corresponding to each subcriterion were collected to construct the decision matrix. Most of the data for these indicators comes from TerriData (Colombia’s official territorial statistical platform managed by the National Planning Department). No missing or inconsistent data were identified, as the databases used originate from official sources that apply standardized methodologies for data collection, validation, and estimation. TerriData integrates sectoral administrative records, Quality of Life Surveys (ECV), and demographic projections from DANE, while also incorporating imputation and estimation procedures based on historical series and population models [34,35]. These practices ensure the consistency and comparability of the information across municipalities, including rural areas with limited availability of administrative records.

Advanced indicators or dynamic data, such as network traffic patterns, user behavior analytics, equity indices, digital inclusion indices, or accessibility factors (which are relevant to cost/benefit analysis) were not used due to their limited or nonexistent availability in municipalities. Table 4 details the data sources utilized.

**Table 4:** Information sources for the selection criteria.

Subcriterion	Document	Source
Number of inhabitants	Territorial Statistics System, Terridata	National Planning Department (DNP), Colombia, 2024
Rural population		
Urban population		
Victims of the Armed conflict		
4G coverage	Coverage Map	Telecommunications Operators in Colombia, 2024
Broadband penetration	Territorial Statistics System, Terridata	National Planning Department (DNP), Colombia, 2024
Rural electricity coverage		
Household telephone service		
Surface area		
Terrain Roughness Index (TRI)	Mathematically computed using a GIS system	QGIS, 2024
Primary sector	Departmental Development Plan 2024-2027	Magdalena Department Government, 2024
Secondary sector		
Hospital network		
Tourism	Territorial Statistics System, Terridata	National Planning Department (DNP), Colombia, 2024

Source: Authors.

In this study, cost-benefit simulation models were not incorporated because, according to guidelines from the Ministry of Information and Communication Technologies (MinTIC) of Colombia, capital expenditure (CAPEX) and operational expenditure (OPEX) values for 5G base station implementation are standardized and fixed nationally [36]. For instance, a base station powered by the commercial grid, with a tower under 50 meters located in an easy-access zone (Type 1), has an estimated initial investment of approximately USD $202000 (as of 2025), with a monthly operational cost of around USD $430, totaling USD $5140 annually. This standardization implies no significant cost variability across municipalities, which limits the utility of economic simulation within a territorial prioritization model. Therefore, the multicriteria analysis focused on technical, sociodemographic, geographic, and service-based dimensions where structural differences are evident.

#### Computation of the Terrain Roughness Index (TRI).

To determine the Terrain Roughness Index (TRI) for each municipality in the Magdalena department, a Digital Elevation Model (DEM) was obtained from the United States Geological Survey (USGS) via EarthExplorer. This platform provides access to digital surface data in raster format, enabling users to search, visualize, and download elevation data. Each cell in the downloaded DEM has a resolution of 30 arc-seconds, equivalent to approximately one kilometer [37].

**Fig 6:**
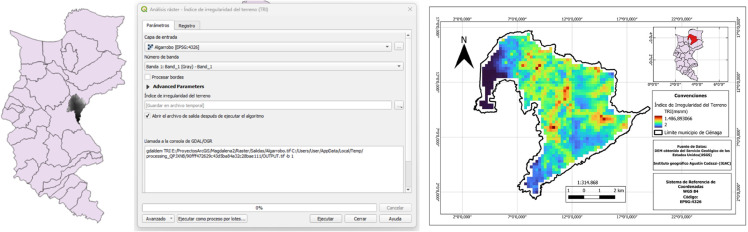
Images of the TRI calculation.

Once the raster data was downloaded, it was clipped for each municipality using the “Clip Raster” algorithm implemented in Quantum GIS (QGIS), utilizing the municipal boundaries obtained from the *“Colombia en Mapas”* platform of the Agustín Codazzi Geographic Institute (IGAC). With the DEM for each municipality, the TRI was then generated using the algorithm implemented in QGIS. Fig 6 graphically illustrates the implementation of the described procedure.

**Fig 7:**
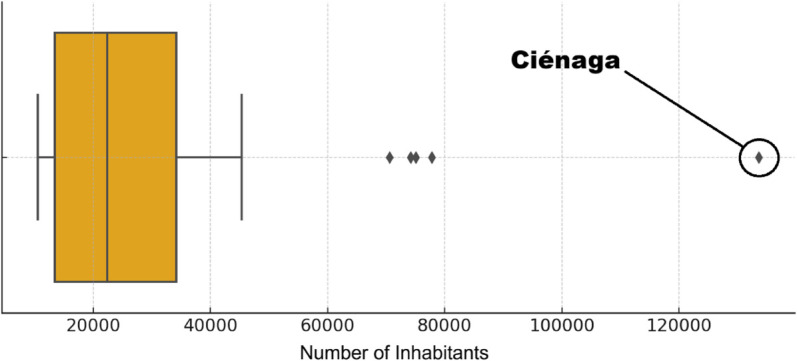
Box plot of the “Number of Inhabitants” criterion.

In the application of the multicriteria analysis, normalization techniques and consistency checks were used to mitigate the disproportionate impact of potential outliers. While outlier analysis may be relevant in some contexts, in our case, the model was built to assess global robustness, not to interpret individual values. The apparent outliers reflect specific territorial realities. This is illustrated in Fig 7, which shows a box-and-whisker plot for the “Number of Inhabitants” criterion, where outliers correspond primarily to the municipality of Ciénaga, with 133601 inhabitants.

### Sensitivity analysis

To evaluate the model’s stability against variations in criterion weights, a sensitivity analysis was performed using the Monte Carlo method. This stochastic simulation approach accounts for uncertainty by generating multiple random scenarios based on predefined probability distributions. In this case, probability distributions were assigned to the criterion weights for each alternative, and 10000 iterations were executed [38–40], where new weight values were generated, and the alternative rankings were recalculated using the TOPSIS and SAW methods.

Subsequently, the results from each iteration were compared to assess the variability in alternative prioritization and the consistency between both methods. Finally, statistical techniques were applied to measure the correlation between the generated rankings, providing a quantitative assessment of the model’s stability and robustness against changes in criterion weights.

For the sensitivity analysis using the Monte Carlo method, the selection of appropriate probability distributions was crucial for generating random values for the solution alternatives. Commonly, uniform, normal, and triangular distributions are used due to their simplicity and interpretability [41–44]. However, in specific contexts, distributions such as LogNormal, Beta, and Gamma provide a more realistic representation of uncertainty in the evaluated criteria.

The LogNormal distribution is suitable for strictly positive variables with a skew toward higher values, such as costs or response times [43,45]. The Beta distribution allows values within a bounded range, making it ideal for modeling performance indicators expressed as proportions or probabilities [46,47]. The Gamma distribution is useful for representing waiting times or positively skewed events, such as system reliability or process duration [48,49].

To identify the most suitable probability distribution for each of the criteria used in the multicriteria model, normality tests (Kolmogorov-Smirnov and Shapiro-Wilk) and goodness-of-fit analyses were conducted against Normal, LogNormal, gamma, and beta distributions. The results shown in Table 5. indicate that most criteria (such as rural population, urban population, broadband penetration, and rural electricity coverage) fit better to a LogNormal distribution, consistent with the strictly positive nature and asymmetry of these data. In the case of the ‘conflict victims’ criterion, a gamma distribution provided the best fit, reflecting a concentration of low values and significant dispersion in higher ones. The selection of each distribution was based on the highest p-value obtained from the Kolmogorov-Smirnov test, which allowed us to not reject the null hypothesis of distribution fit. Notably, the criterion “Number of Inhabitants” is the sum of “Rural Population” and “Urban Population” and is therefore not included in Table 5.

**Table 5:** Comparison matrix of economic activity subcriteria.

Criterion	Distribution	Statistic	P-value
Rural Population	LogNormal	0.1045	0.8765
Urban Population	LogNormal	0.1292	0.6706
Victims of the Armed Conflict	Gamma	0.1818	0.2598
4G Coverage	Binomial	NA	NA
Broadband Penetration	LogNormal	0.0875	0.9651
Rural Electricity Coverage	LogNormal	0.1525	0.6046
Telephone Service in Households	LogNormal	0.1652	0.3664
Surface Area	LogNormal	0.0688	0.9975
Terrain Roughness Index	LogNormal	0.1783	0.2799
Primary Sector	LogNormal	0.0993	0.9101
Secondary Sector	LogNormal	0.1394	0.4741
Hospital Network	Binomial	NA	NA
Tourism	LogNormal	0.1371	0.9226

Source: Authors.

### Dependency analysis

The dependency analysis sought to assess whether the presence of highly correlated criteria could affect the prioritization results. To this end, a complementary approach to the previously described AHP was applied, integrating the objective CRITIC method along with tests excluding redundant criteria. This stage allowed the comparison of weights derived from expert judgments with those calculated based on the statistical structure of the data.

The CRITIC method (Criteria Importance Through Intercriteria Correlation) objectively assigns weights to criteria by considering both their variability and their level of correlation with others. Thus, criteria that provide more information and exhibit lower redundancy receive greater weight, while highly correlated ones lose relevance [50,51]. Unlike AHP, which relies on pairwise comparisons made by experts, CRITIC dispenses with subjective judgments and relies solely on statistical properties of the data. In this way, it offers a complementary perspective that helps validate the robustness of the prioritization model.

Based on this information, CRITIC was applied to calculate objective weights, which were then used in the evaluation of alternatives through TOPSIS and SAW. The results were compared with the original rankings obtained using AHP-TOPSIS and AHP-SAW. Additionally, a test scenario was designed in which the most highly correlated criteria were excluded. The AHP weights were redistributed among the remaining criteria, and the rankings were recalculated. This variant made it possible to evaluate how information redundancy could influence the stability of the model and to contrast the results against both the original AHP and the CRITIC-based approach. The final step consisted of comparing all generated rankings (AHP-TOPSIS, AHP-SAW, CRITIC-TOPSIS, CRITIC-SAW, and the adjusted AHP without redundant criteria).

## Results

The most relevant findings of this research are presented below.

### Municipality information

One of the key outcomes of this study is the identification of subcriteria and the sociodemographic, geographic, economic, and technological factors that support the prioritization of municipalities for the early deployment of the 5G network. Additionally, the collected data is presented and organized by municipality, detailing the values associated with each subcriterion.

S1 Table compiles the subcriterion data for each municipality. Moreover, these subcriteria are classified as either cost (-) or benefit (+). Benefit criteria are those where higher values are preferable, whereas cost criteria are those where lower values are desirable. This classification is crucial as it determines how the data is normalized and how distances to the ideal and anti-ideal solutions are calculated in the TOPSIS analysis.

### Weighting of criteria and subcriteria

To determine the weights of the criteria and subcriteria, responses from eight experts were analyzed. These experts, representing both academia and the productive sector, have extensive experience in telecommunications and territorial transformation. The Consistency Ratio (CR) was calculated for each expert to validate their responses, along with a global CR for each criterion and subcriterion.

S1 File presents the CR values, preference scores, and calculated weights of the criteria and subcriteria. Initially, the experts’ evaluations were aggregated using geometric means. Subsequently, the criterion weights were determined, followed by the computation of the local and global weights for the subcriteria. The global weight was obtained by multiplying the local weight by the corresponding criterion weight.

The results indicate that sociodemographic and technological factors have the greatest impact on planning the deployment of 5G networks. Additionally, the most relevant subcriteria are the number of inhabitants, land area, and the existence of 4G network coverage in municipalities, with respective weights of 13.13%, 12.16%, and 11.13%.

**Fig 8:**
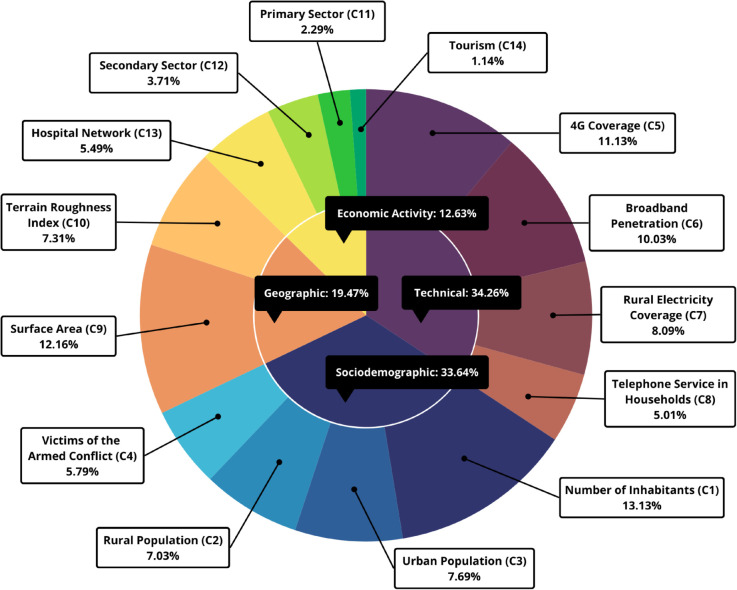
Weights of selection criteria and subcriteria.

Fig 8 presents the global weighting results for each criterion, which are subsequently used for prioritization with TOPSIS and SAW.

### Ranking of municipalities

**Fig 9:**
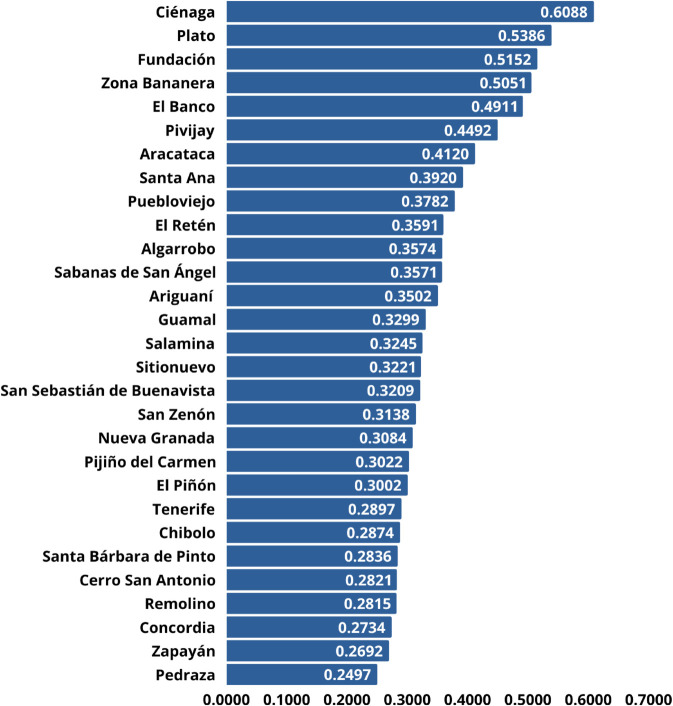
Ranking of municipalities for 5G network deployment using AHP-TOPSIS.

Fig 9 presents the ranking of municipalities in the Magdalena department, Colombia, prioritized for 5G network deployment based on their distance to the ideal solution using the TOPSIS method. The corresponding proximity percentage is also included.

**Fig 10:**
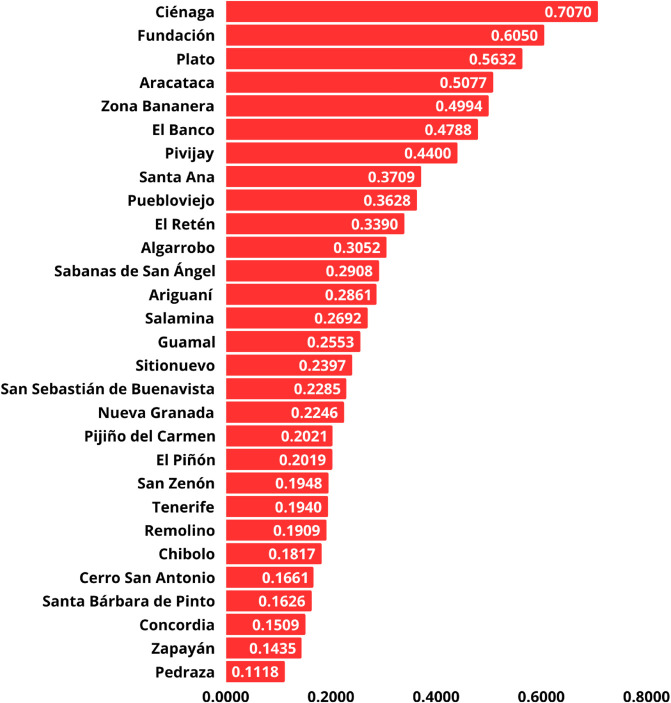
Ranking of municipalities for 5G network deployment using AHP-SAW.

Similarly, Fig 10 displays the ranking of municipalities prioritized for 5G network deployment using the SAW method. The weighted score of each alternative is provided.

### Model stability

**Fig 11:**
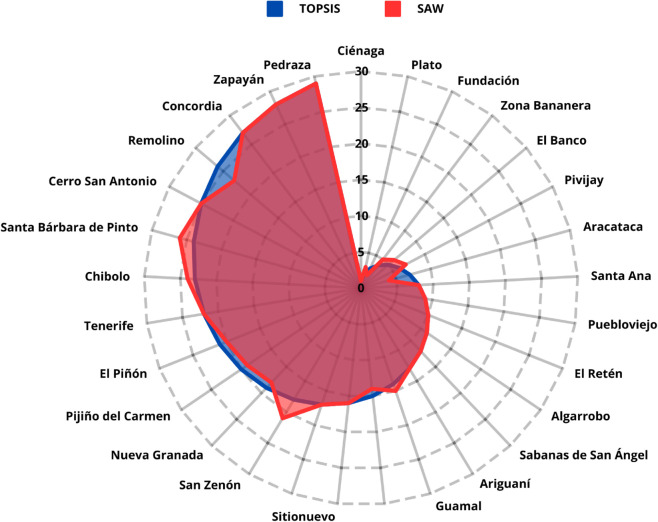
Comparison of results obtained by TOPSIS and SAW.

A Spearman’s rank correlation coefficient of *ρ* = 0.9897 (p-value < 0.0001) was obtained when comparing the rankings generated by the TOPSIS and SAW methods. Both approaches ranked the municipality of Ciénaga in first place, while Fundación and Plato alternated between second and third positions. Additionally, Zona Bananera occupied either the fourth or fifth position, depending on the method used. This strong and statistically significant correlation indicates a high degree of consistency between the two methods, suggesting that the proposed prioritization is robust. Fig 11 graphically compares the rankings obtained by TOPSIS and SAW.

**Fig 12:**
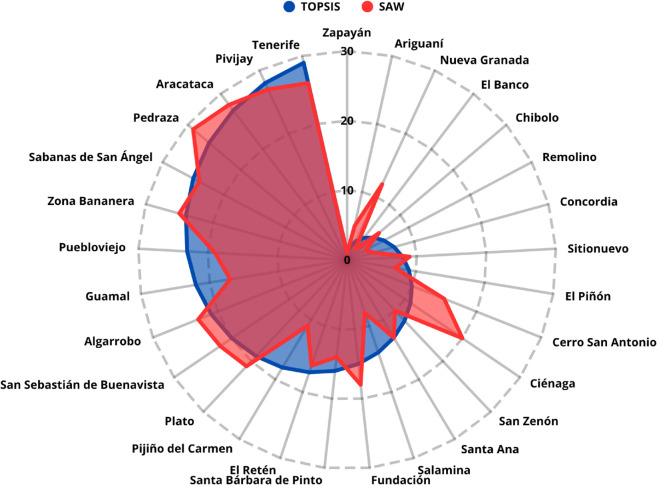
Results obtained using the Monte Carlo method.

After executing a total of 10000 iterations, a Spearman’s rank correlation coefficient of *ρ* = 0.9010 was obtained between the TOPSIS and SAW results, with a p-value < 0.0001. This indicates a statistically significant relationship between both methods, confirming the consistency of the rankings despite variations in criterion weights. Fig 12 presents a comparison of the results obtained for each multicriteria decision-making method.

**Fig 13:**
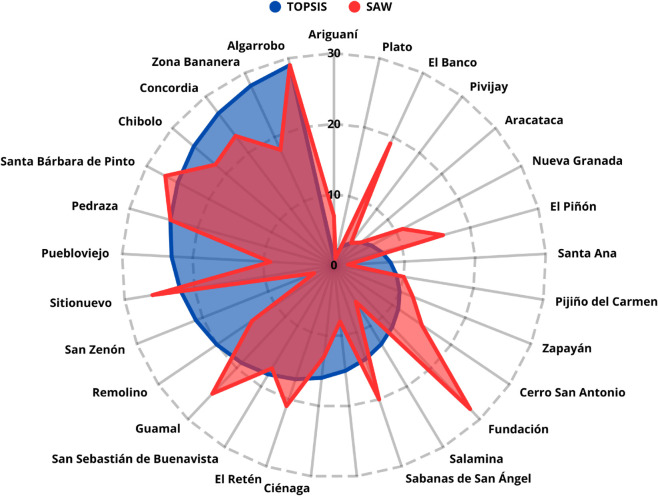
Impact of excluding main subcriteria on model robustness.

As a complementary analysis, a sensitivity test was conducted by excluding the three subcriteria with the highest global weights—Number of Inhabitants, Surface Area (km^2^), and 4G Coverage—which together account for approximately 36% of the total weighting. Under this scenario, when applying the TOPSIS and SAW methods, the correlation between the rankings decreased to 0.6020 (p-value < 0.0001), as shown in Fig 13. This indicates a significant reduction in the robustness of the model. The result confirms that these subcriteria are critical to the stability of prioritization and that their exclusion introduces greater uncertainty in the comparison of alternatives.

### Validation of the 5G deployment prioritization model against the MinTIC population-based approach

**Fig 14:**
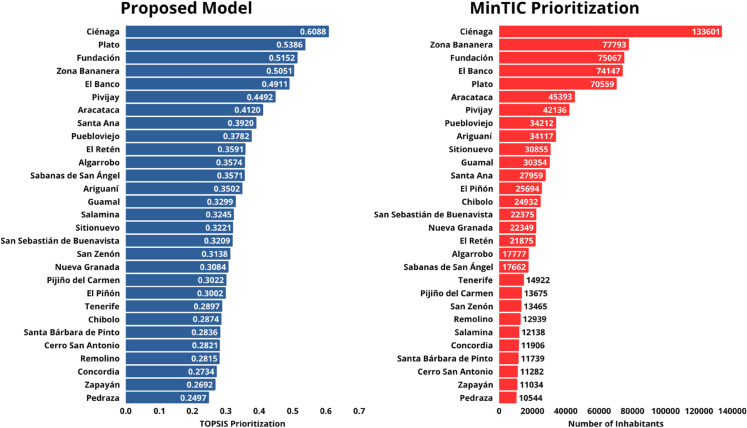
Comparison of the MinTIC prioritization and the TOPSIS method used.

Following the model proposed by the MinTIC of Colombia, which considers only the number of inhabitants as a prioritization criterion for 5G deployment , a correlation of *ρ* = 0.8714 (p-value < 0.0001) was obtained between the ranking generated by MinTIC and the one generated by our proposed model. Fig 14 presents a comparison of both results, showing the effectiveness of our model by reaching a correlation above 85

This supports the conclusion that the population size (a criterion used both by MinTIC and our model) is indeed relevant. However, we recommend incorporating additional variables for prioritization, especially in municipalities with similar population sizes.

### Correlation analysis between selection criteria

**Fig 15:**
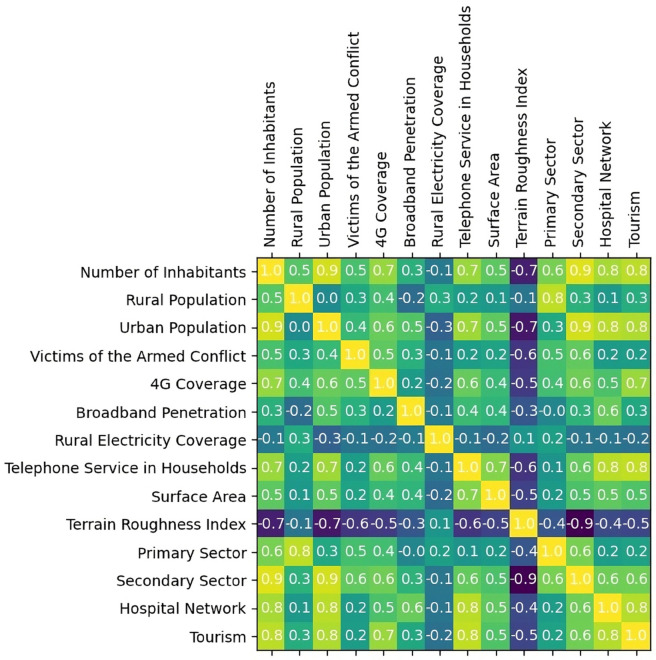
Correlation matrix between selection criteria.

Fig 15 shows a high positive correlation between the number of inhabitants and the urban population (*ρ* = 0.9), as well as between the latter and the secondary sector (*ρ* = 0.9), suggesting that industrial activity is more likely to be found in urbanized contexts. Similarly, 4G coverage exhibits relevant positive correlations with demographic and infrastructure-related variables such as urban population (*ρ* = 0.6), in-home telephone service (*ρ* = 0.7), and tourism (*ρ* = 0.7), reflecting a common pattern of technological resource concentration in densely populated areas.

Conversely, the Terrain Roughness Index shows significant negative correlations with several key variables: number of inhabitants (*ρ* = –0.7), urban population (*ρ* = –0.6), 4G coverage (*ρ* = –0.5), and hospital network (*ρ* = –0.5). This evidence suggests that unfavorable geographical conditions tend to limit demographic and technological development, introducing structural relationships between criteria that cannot be considered mutually independent.

### Evaluation of dependency between criteria and its impact on prioritization

Following the correlation analysis of the criteria, a comparative exercise was conducted to assess how the prioritization results varied when applying objective methods and when adjusting the model through the exclusion of highly correlated criteria. To this end, the rankings generated with AHP–TOPSIS and AHP–SAW were contrasted with their equivalents derived from CRITIC-based weightings, as well as with scenarios in which AHP weights were redistributed after removing redundant criteria.

**Fig 16:**
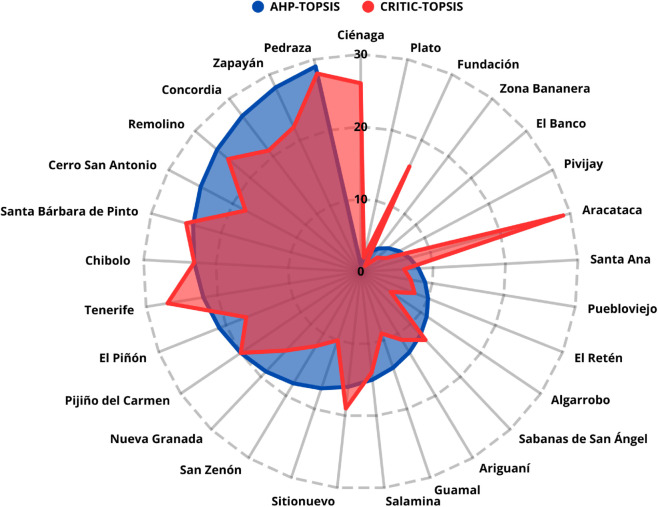
Comparison between AHP–TOPSIS and CRITIC–TOPSIS rankings.

**Case 1. Comparison AHP–TOPSIS vs. CRITIC–TOPSIS:** When applying the CRITIC method with all criteria and subcriteria and contrasting it with AHP–TOPSIS, a correlation of 0.4886 with p-value < 0.0001 was obtained, reflecting a substantial difference between the rankings. This result shows that introducing an objective weighting method significantly alters the order of prioritization, as presented in Fig 16.

**Fig 17:**
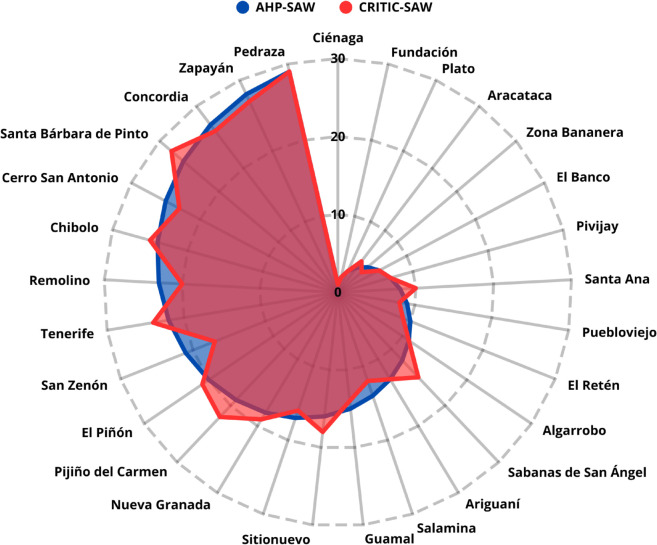
Comparison between AHP–SAW and CRITIC–SAW rankings.

**Case 2. Comparison AHP–SAW vs. CRITIC–SAW:** The contrast between AHP–SAW and CRITIC–SAW yielded a correlation of 0.9879 with p-value < 0.0001, indicating a high degree of similarity between the rankings, as shown in Fig 17. This finding suggests that, in this scenario, the weights derived from expert judgment and those calculated objectively produce consistent results.

**Fig 18:**
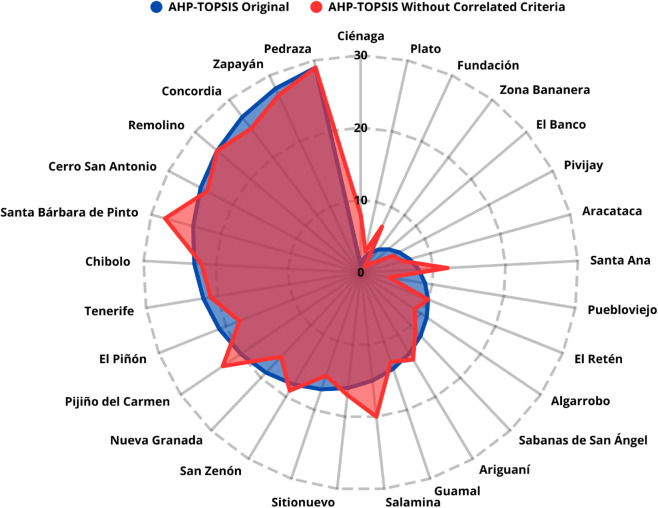
Comparison between AHP–TOPSIS rankings with and without correlated criteria.

**Case 3. Adjusted scenario AHP–TOPSIS without highly correlated criteria:** By excluding the most correlated criteria (Number of Inhabitants, Urban Population, Secondary Sector, and Tourism), the AHP–TOPSIS model yielded a correlation of 0.7619 with p-value < 0.0001, relative to the original prioritization. This demonstrates that removing redundancies partially modifies the results, revealing the sensitivity of the ranking to dependency between criteria, as shown in Fig 18.

**Fig 19:**
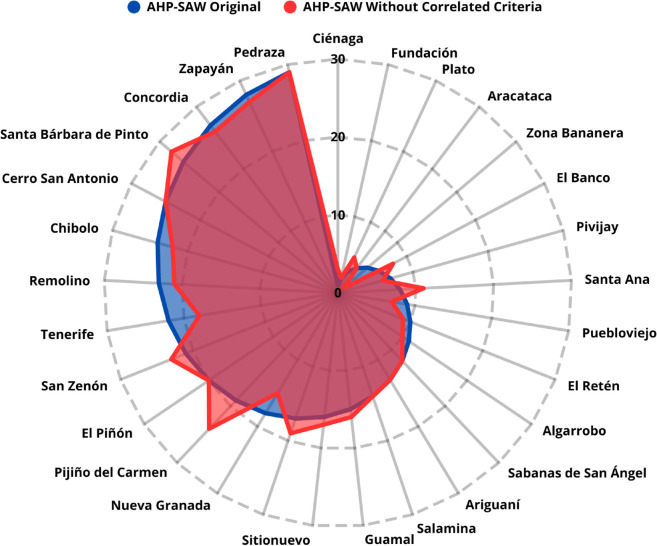
Comparison between AHP–SAW rankings with and without correlated criteria.

**Case 4. Adjusted scenario AHP–SAW without highly correlated criteria:** In the same exclusion scenario, the AHP–SAW model achieved a correlation of 0.9618 with p-value < 0.0001, relative to the original ranking, as presented in Fig 19. In this case, the variation is smaller than in the TOPSIS scenario, indicating greater stability of the results when the SAW method is applied.

## Discussion

This study presents a robust methodology for prioritizing 5G network deployment using a multicriteria approach that integrates technical, sociodemographic, geographic, and economic factors. The following discussion contextualizes the main findings within telecommunications infrastructure planning while addressing potential limitations of the proposed model.

Technical and sociodemographic criteria emerged as the most influential factors in selecting municipalities for 5G implementation. This suggests that infrastructure expansion prioritizes efficiency and return on investment, favoring areas with higher population density and existing telecommunications infrastructure. However, this strategy may disproportionately benefit urban and semi-urban areas, leaving rural communities with lower commercial appeal underserved. This finding underscores the need for complementary public policies to ensure equitable technological access, preventing the 5G rollout from exacerbating the digital divide.

In Colombia, official indicators on the digital divide are reported at the departmental level, not the municipal one, according to the National Planning Department (DNP). This limitation requires indirect approaches to assess intra-departmental inequalities, as done in our multicriteria model, which integrates socioeconomic, infrastructure, and digital vulnerability variables from official sources such as TerriData. These include 4G coverage, broadband penetration, in-home phone access, and rural electricity coverage. Thus, our methodological proposal provides a useful tool to prioritize actions at the intra-departmental level within the digital divide context. According to the 2023 digital divide index published by the MinTIC, the Magdalena department shows a divide 18% higher than the national average [52].

The sensitivity analysis using the Monte Carlo method confirms the model’s stability against variations in criterion weights, suggesting its applicability in different geographic and economic contexts. However, a key limitation is that the model does not account for market dynamics, specific government regulations, or private investment strategies, all of which can significantly impact deployment feasibility. These factors were excluded based on the study’s scope, which prioritizes municipalities beyond the contractual obligations and investment incentives of the frequency band auction. Future research should integrate variables such as government incentives, implementation costs, public-private partnerships, real-time connectivity demand, mobile network usage, and financial feasibility assessments for 5G deployment in prioritized municipalities.

In this regard, the present research provides a methodological foundation for future developments that incorporate forecasting tools such as ARIMA models and Artificial Intelligence and Machine Learning techniques, including Long Short-Term Memory (LSTM) networks. These approaches enable the processing of information flow by retaining relevant data over extended periods and prioritizing critical elements for predicting future sequences [53,54]. Their integration would facilitate the inclusion of dynamic variables such as projected demand growth, traffic load estimation, and usage behavior analysis in both urban and rural scenarios.

Although TOPSIS and SAW produced highly correlated rankings (*ρ* = 0.9897), confirming the model’s consistency, the choice of decision-making method may influence final prioritization. Each method has its strengths and limitations: TOPSIS favors alternatives that balance multiple criteria, while SAW weights the sum of normalized values, potentially amplifying the influence of certain subcriteria. Likewise, the comparison between the rankings obtained with AHP-SAW and AHP-TOPSIS, those generated after excluding redundant criteria, and the objective methods CRITIC-SAW and CRITIC-TOPSIS, made it possible to assess whether the presence of highly correlated criteria could affect prioritization results. Consequently, infrastructure deployment decisions may be affected by the evaluation technique used, emphasizing the importance of applying multiple approaches to validate results.

In addition, executives from major telecommunications operators in Colombia were consulted, who confirmed that there is no public evidence that municipalities such as Ciénaga and Fundación are prioritized by the National Spectrum Agency (ANE) for 5G deployment. Likewise, academic experts, representatives from the productive sector, and professionals involved in territorial transformation projects were consulted, seven out of eight of whom agreed that the model’s prioritization is consistent. This dual exercise—combining institutional review and expert feedback—strengthens the practical validity and credibility of the multicriteria model, even in the absence of empirical validation in the field.

The correlation analysis revealed potential associations between certain criteria, which may affect the independence assumption that supports the AHP method. Although the current framework maintained this assumption for consistency and simplicity, future applications in other regions should incorporate a preliminary correlation assessment to determine whether a more suitable method (such as the Analytic Network Process (ANP) or objective weighting approaches like CRITIC or entropy-based weighting) is warranted to account for interdependencies [32,55].

Although there are multicriteria methods such as PROMETHEE, VIKOR, and ELECTRE that are useful for evaluating alternatives across multiple criteria, this research opted for AHP combined with TOPSIS and SAW due to its clearer methodology and ease of integrating expert judgment via pairwise comparisons. PROMETHEE and ELECTRE are powerful and valid methods, but their interpretation may be less intuitive for decision-makers unfamiliar with concepts like net preferences or dominance relations [56,57]. In contrast, the AHP-TOPSIS-SAW combination allows for transparent and verifiable hierarchical prioritization, suitable for institutional and public planning contexts.

This work was conceived as an exploratory technical and quantitative study focused on the development of a decision-support tool for planning 5G network deployment in the Department of Magdalena. Unlike regulatory or public policy proposals, the developed model seeks to guide operators, engineers, and technical stakeholders in territorial prioritization through a systematic and reproducible approach. This scope reflects the intention to preserve the technical neutrality of the study and to acknowledge that administrative and regulatory decisions are the responsibility of competent government authorities.

Accordingly, the priority of this study was not to formulate specific political or administrative recommendations but to ensure the robustness of the model in the face of structural variability in territorial conditions. The implementation of a sensitivity analysis through Monte Carlo simulations allowed for validation of the ranking’s stability against changes in criterion weights, which reinforces the framework’s utility in contexts of high uncertainty or pronounced regional disparities. This approach provides a solid foundation for replication in other regions, as long as proper contextual adaptation is ensured.

## Conclusion

The proposed model effectively prioritizes municipalities for 5G network deployment by integrating technical, sociodemographic, geographic, and economic factors within a structured AHP framework. The strong correlation between TOPSIS and SAW rankings suggests the model’s replicability, while Monte Carlo sensitivity analysis confirms its stability against variations in criterion weights.

A key finding is that technical (34.26%) and sociodemographic (33.64%) factors play the most significant roles in prioritization, with geographic (19.47%) and economic (12.63%) criteria also exerting considerable influence. In the case study, Ciénaga emerged as the highest-priority municipality for 5G deployment, given its extensive 4G coverage, large urban population, and strong economic activity in the secondary sector. These factors enable efficient service provision, requiring fewer base stations while leveraging existing 4G LTE infrastructure (Non-Standalone). Additionally, its well-developed hospital network and tourism potential ensure sustained demand for 5G services. More broadly, municipalities with larger populations and established telecommunications infrastructure have a competitive advantage, though economic activity and topography remain relevant considerations.

The complementary analysis showed that excluding the subcriteria with the highest global weights (Number of Inhabitants, Surface Area, and 4G Coverage), which together account for 36% of the total weighting, reduced the correlation between TOPSIS and SAW to 0.6020, highlighting the model’s sensitivity. Similarly, when highly correlated criteria (Number of Inhabitants, Urban Population, Secondary Sector, and Tourism) were removed, AHP–TOPSIS yielded a correlation of 0.7619 with respect to the original model, while AHP–SAW maintained greater stability (0.9618). Finally, the comparison with CRITIC revealed substantial divergences in TOPSIS (0.4886) and a high similarity in SAW (0.9879), confirming that the model’s consistency depends both on the method applied and on the degree of independence among the criteria.

Thanks to the observed stability in the consistency between the AHP-TOPSIS and AHP-SAW models, the results indicate that municipalities with lower telecommunications development and weaker sociodemographic indicators are deprioritized, potentially widening the region’s digital divide. To address this, future public policies should introduce incentives for infrastructure investment in less commercially attractive areas, ensuring more equitable 5G coverage aligned with digital inclusion goals.

## Supporting information

S1 Table Municipality information. (XLSX)

S1 File CR values, preference scores, and calculated weights of the criteria and subcriteria. (XLSX)
